# Cryoablation Catheter Used in the Surgical Treatment of Atrial Fibrillation May Treat Chest Tube Pain: Engin Technique

**DOI:** 10.21470/1678-9741-2023-0354

**Published:** 2024-05-13

**Authors:** Mesut Engin, Ufuk Aydın, Ahmet Kağan AS, Yusuf Ata, Şenol Yavuz

**Affiliations:** 1 Department of Cardiovascular Surgery, University of Health Sciences, Bursa Yuksek Ihtisas Training and Research Hospital, Bursa, Turkey

**Keywords:** Chest Tubes, Pain, Postoperative Period, Cryosurgery

## Abstract

Postoperative pain after cardiac surgery plays an important role in the patient’s recovery process. In particular, pain at the chest tube site can negatively affect the comfort and recovery of these patients. Effective pain control minimizes the risk of many complications. Oral and intravenous analgesics, epidural anesthesia, paravertebral block, and intercostal nerve blockade are used in chest tube pain control. We routinely use the surgical cryoablation method in the presence of atrial fibrillation in the preoperative period of cardiac surgery in our clinic. Here we aimed to describe our method of using the cryoablation catheter for intercostal nerve blockade.

## INTRODUCTION

Postoperative pain after cardiac surgery plays an important role in the recovery process of the patient. In particular, pain at the chest tube site can negatively affect the comfort and recovery of patients in the postoperative period^[1]^. Therefore, effective pain control to be applied to this patient group will also minimize the risk of many complications^[2,3]^. Oral and intravenous analgesics, epidural anesthesia, paravertebral block, and intercostal nerve blockade are used in chest tube pain control^[4]^.

We routinely use the surgical cryoablation method in the presence of atrial fibrillation in the preoperative period of cardiac surgery in our clinic. In this technical presentation, we aimed to describe our method of using the cryoablation catheter for intercostal nerve blockade.

## TECHNIQUE

A 56-year-old man was referred to our cardiac surgery team for consideration of mitral valve replacement for severe mitral insufficiency. He had normal coronary arteries, and his electrocardiogram demonstrated atrial fibrillation. His medical history is notable only for hypertension. Written informed consent was obtained. The patient was scheduled for cryoablation and surgical mitral valve replacement through a full sternotomy.

**Fig. 1:**
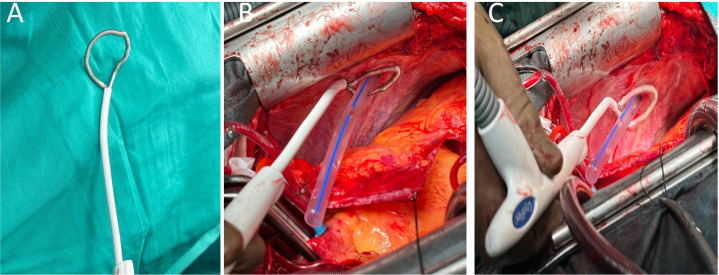
(A) The flexible catheter was positioned, (B) the catheter was placed around the previously placed chest tube in contact with the tissue, and (C) a single 90-second freeze cycle was performed at 60% power, to ensure the temperatures were between -60°C and -100°C.

After the patient was induced with general endotracheal intubation, he was prepped in the usual standard fashion. A median sternotomy was performed. Standard cardiopulmonary bypass was utilized with mild hypothermia (32°C). A cross-clamp was placed to the ascending aorta, and cardiac arrest was provided. Then mitral valve replacement was performed with a left atrial approach, and left atrial surgical cryoablation was applied with the Cardioblate® CryoFlex™ Surgical Ablation Probe (Medtronic Inc., Minneapolis, Minnesota, United States of America). After the cardiac surgical procedures were completed, the cryoablation catheter was used again for the chest tube site. First, the flexible catheter was positioned (Figure 1A). The catheter was placed around the previously placed chest tube in contact with the tissue (Figure 1B). A single 90-second freeze cycle was performed to ensure the temperatures were between -60°C and -100°C (Figure 1C).

After the surgical procedures, the patient was transferred to the intensive care unit. He was extubated on the fourth postoperative hour. Chest tube pain assessments (visual analog scale) of the patient in the postoperative period were made at the sixth hour, 24th hour, 36th hour, and after tube extraction. Pain ratings were 0, 1, 1, and 2, respectively. The patient was discharged on the seventh postoperative day. The patient was readmitted to the clinic one week postoperatively. His sternal incision was well healed, and the sternum was stable. Sensation at the chest tube site had begun to return with no pain.

## DISCUSSON

Here, we showed that the cryoablation catheter, which was used in the surgical treatment of atrial fibrillation in the literature, can also be used for chest tube pain. Chest tube pain can cause various problems, especially atelectasis, after cardiac surgery. Various methods have been described to prevent this pain, such as oral or parenteral use of various painkillers, and blockade with local anesthetic agents^[2-4]^. In addition, many methods have been studied to relieve pain during chest tube removal^[5]^. Our method also shows its effect on this problem.

Cryoablation can be used for thoracotomy pain^[6,7]^. In addition, the thoracic cryoablation method was described by Caparrelli for the management of postoperative analgesia in a patient who underwent cardiac surgery with full sternotomy^[8]^.

### Limitation

The method described here is applicable to a limited number of patients in terms of cost and effectiveness.

## CONCLUSION

Cryoablation may be an effective method for postoperative chest tube pain in cardiac surgery patients who will also undergo cryoablation due to atrial fibrillation. It can be considered for pain control in all appropriate patient groups.
